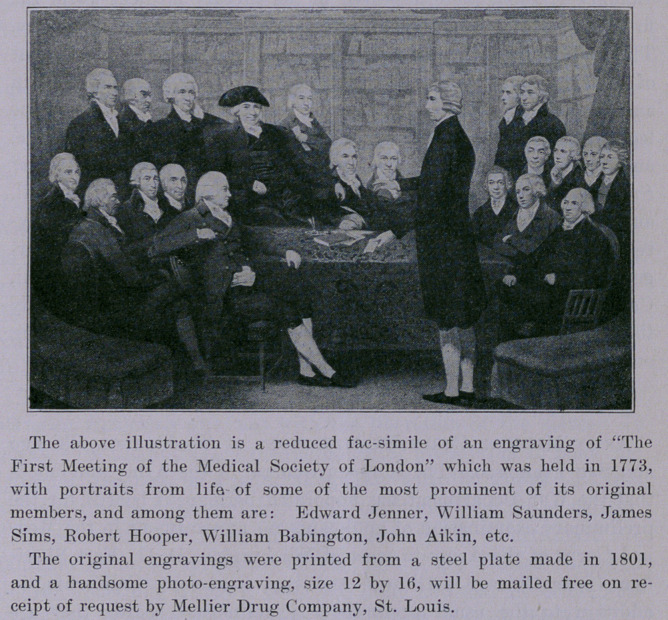# Editorialets

**Published:** 1909-12

**Authors:** 


### Editorialets.

Evolution of the Great Medical Trust (Chronicles of The Octopus) is republished in this issue of the Journal, in response to numerous requests for copies, and I have added "The Fly in the Ointment," "Song of the First Lord," and "Lifting of the Veil," in order that I may have reprints made of them. They will be published in pamphlet form, embellished with a cut of the now famous Lincoln Medical Institute and Water Cure Establishment which was being conducted by Dr. Simmons and advertised in the *Nebraska State Journal* (a newspaper) when he was discovered in the early eighties. These pamphlets can be had for $1 a dozen, or 10 cents a copy.

---

Dr. E. H. Lancaster, State Bacteriologist of Texas, was married at Austin, December 2, to Ellen, daughter of the late Professor and Mrs. Leslie Waggener.

---

I do not want to miss a copy of the "Red Back." It is undoubtedly the live wire of Texas medical journalism. Long may it live. Sincerely, W. A. Bedford, M. D., Thornton, Texas.

---

Dr. Geo. H. Moody, of San Antonio, was elected President of the Medical Association of the Southwest at the great meeting recently held in San Antonio. The Association honored itself in this selection.

---

My Subscribers who are in arrears will all receive their bills this month. I hope they will all remit (personal checks will be accepted), otherwise Betty and the baby will have no Christmas turkey and Santa Claus will give them the shake. Do it now.

---

For Sale.—A $2500 unopposed practice in a thickly settled Bohemian and German community; collections average 98 per cent; will sell residence and stock of drugs for $1700; part cash and balance on easy payments, if desired. Address Dr. S., care of Texas Medical Journal.

---

Philadelphia Number.—*The American Journal of Surgery* will produce in December a Philadelphia issue of their journal, the subject matter of which will be composed entirely of contributions from among the leading men of that city. Among the contributors to this issue are Montgomery, Adler, Hirst, Davis, Pyle, Leonard, Hitschler, Bland, La Place, Posey, Clark, Christian, McGlinn *et al.*

Dr. Lydston denounces as false and purposely misleading the garbled "interview" published in a St. Louis paper. Such methods are despicable and contemptible, and show the desperate straits friends of the great MISMANAGEMENT are in to save the Bosses. Such transparent mendacity will not go in the South or prejudice Southerners against the brave defender of principle. Lydston was the first to advocate castration for rape.

---

The above illustration is a reduced fac-simile of an engraving of "The First Meeting of the Medical Society of London" which was held in 1773, with portraits from life of some of the most prominent of its original members, and among them are: Edward Jenner, William Saunders, James Sims, Robert Hooper, William Babington, John Aikin, etc.

The original engravings were printed from a steel plate made in 1801, and a handsome photo-engraving, size 12 by 16, will be mailed free on receipt of request by Mellier Drug Company, St. Louis.

---

The Hook Worm.—Seven years have elapsed since both the cause and the remedy became known and no steps could be taken for the extermination of this parasite—fittingly named by Dr. Stiles *Necator Americanus*—"the American murderer"—because there seems to be no law authorizing the expenditure of money by the national government for this purpose. How different would it have been had the lives and health of a few million horses, cattle or sheep been involved instead of merely a few million human beings! Nothing could better illustrate the need of a bureau of public health, authorized and equipped to carry out at least an aggressive campaign of education regarding the cause, prevalence and remedy of such diseases.—*Exchange.*

Seeing this, the much abused philanthropist and humanitarian, John D. Rockefeller, steps to the front and pours out at Dr. Stiles' feet—or Uncle Sam's—one million dollars!

---

THE ANNALS OF SURGERY ISSUES ITS FIFTIETH VOLUME.—On January 1, 1885, there appeared in the literary medical world the first number of a new journal, given up entirely to general surgery. This radical departure from the old lines had the full endorsement of a large number of the leaders in surgery, both in Great Britain and the United States, among whom was Lord Lister, whose name led all the rest on the title-page. The seed was good, the soil fertile, and the journal grew and prospered. Today it's the *Annals of Surgery,* of Philadelphia. In December it blooms—blooms in full, and its subscribers will be treated to a choice collection of twenty-two original articles in the form of a jubilee number. Eminent surgeons from England, Scotland, Denmark, France, Italy, Hawaii, Canada and the United States will contribute to this issue. Truly the editors and publishers deserve great praise for so fitly rounding out this the fiftieth volume.

---

ARMY MEDICAL CORPS EXAMINATIONS at Washington, Chicago and San Francisco. The Surgeon General of the Army announces that the War Department has appointed permanent boards for the preliminary examination of applicants for appointment in the Medical Corps of the Army to meet at Washington, D. C., Fort Sheridan (near Chicago), Illinois, and San Francisco, California, in addition to the usual preliminary examination boards that are assembled at various army posts throughout the United States from time to time. The permanent boards will hold sessions on the second Monday of each month. A limited number of successful candidates will be appointed first lieutenants in the Medical Reserve Corps (salary $2000 per annum) and assigned to army posts until the next session of the Army Medical School, when they will be ordered to attend the school as "student candidates." Applicants must be citizens of the United States, between 22 and 30 years of age, graduates of reputable medical schools, of good moral character and habits, and shall have had a year's hospital training after graduation, or its equivalent. Full information concerning the examination can be procured upon application to the "Surgeon General, United States Army, Washington, D. C."

## Figures and Tables

**Figure f1:**